# SARS-CoV-2 and Variant Diagnostic Testing Approaches in the United States

**DOI:** 10.3390/v13122492

**Published:** 2021-12-13

**Authors:** Emmanuel Thomas, Stephanie Delabat, Yamina L. Carattini, David M. Andrews

**Affiliations:** 1 Sylvester Comprehensive Cancer Center, University of Miami Miller School of Medicine, Miami, FL 33136, USA; sxd788@miami.edu; 2 Schiff Center for Liver Disease, University of Miami Miller School of Medicine, Miami, FL 33136, USA; 3 Department of Pathology, University of Miami Miller School of Medicine, Miami, FL 33136, USA; y.carattini@miami.edu (Y.L.C.); dandrews@med.miami.edu (D.M.A.)

**Keywords:** SARS-CoV-2, COVID-19, PCR, antigen, antibody, nucleic acids

## Abstract

**Purpose of Review** Given the rapid development of diagnostic approaches to test for and diagnose infection with SARS-CoV-2 and its associated variants including Omicron (B.1.1.529), many options are available to diagnose infection. Multiple established diagnostic companies are now providing testing platforms whereas initially, testing was being performed with simple PCR-based tests using standard laboratory reagents. **Recent Findings** Additional testing platforms continue to be developed, including those to detect specific variants, but challenges with testing, including obtaining testing reagents and other related supplies, are frequently encountered. With time, the testing supply chain has improved, and more established companies are providing materials to support these testing efforts. In the United States (U.S.), the need for rapid assay development and subsequent approval through the attainment of emergency use authorization (EUA) has superseded the traditional arduous diagnostic testing approval workflow mandated by the FDA. Through these efforts, the U.S. has been able to continue to significantly increase its testing capabilities to address this pandemic; however, challenges still remain due to the diversity of the performance characteristics of tests being utilized and newly discovered viral variants. **Summary** This review provides an overview of the current diagnostic testing landscape, with pertinent information related to SARS-CoV-2 virology, variants and antibody responses that are available to diagnose infection in the U.S.

## 1. Introduction

Viruses can cause acute or chronic infection with acute infections subsequently cleared by effective host innate and adaptive immune responses [1]. Coronaviruses predominantly cause acute infection [2]; however, cases of “long COVID” have been described mainly in immunocompromised patients [3]. During acute infection, they can possibly cause mortality [4], but most viral infections are cleared and do not establish a persisting chronic infection. The immune response can provide protection against exposure to the same virus to prevent clinically significant reinfection; however, this may not be the case in some patients [5] and also for some viral variants [6]. As with vaccinations or natural infection, the degree of subsequent protection will depend on the length of time after the initial exposure since immune responses wane with time [7] and the degree to which new variants are able to bypass immune responses from previous vaccination and or infection. In general, with acute viruses, individuals may become infected again only after a long interval, but usually the severity of the infection is limited [8,9]; however, new viral variants, that may be more transmissible, are challenging this paradigm. The degree of protection can also depend on the degree of any antigenic shift between the virus that caused the first infection when compared to variants that are responsible for subsequent infections [10].

Given this background, testing for SARS-CoV-2 predominantly relies on testing for evidence of active infection through the detection of viral nucleic acids or viral antigens, whereas chronic infections can most easily, at a reduced cost, be initially detected by the presence of antibodies targeting viral proteins [11,12]. SARS-CoV-2 testing will have to be expanded to adequately address the pandemic and stop the continuing periodic rise of infections. Some estimate that the United States (U.S.) will require testing 3 to 4 million individuals per day to adequately address the pandemic; however, there are only approximately 1 to 2 million individuals tested per day at this time with this information accessed on the 23 of November, 2021 (https://coronavirus.jhu.edu/testing). This is underscored by the fact that SARS-CoV-2 has become endemic to some regions and healthcare facilities within the U.S. [13], and new viral variants are rapidly emerging and replacing previous strains.

Given the rapid development of diagnostic approaches to test for SARS-CoV-2, testing has become much more robust, with more options available to assess infection, identify the presence of distinct viral variants and subsequently prevent virus spread. Multiple established diagnostic companies are now providing testing platforms including Cepheid, Genmark, Hologic, Roche and Abbott [14], whereas initially, testing was being done with simple molecular PCR-based tests using standard laboratory reagents. In addition to challenges with obtaining adequate testing reagents, testing can also be limited by the lack of other supplies including personal protective equipment (PPE), nasal swabs and associated testing reagents, including viral transport media (VTM) [15]. Since the start of the pandemic, the testing supply chain has improved, and more companies are providing materials and products to support these testing efforts that are desperately needed. At this time, the increase in the number of new testing platforms appears to be an additive process due to the high demand to increase testing volume as opposed to a competitive process where performance and cost dictate the use of a specific testing platform. It is important to keep in mind that the results of any test for SARS-CoV-2 will only be accurate based on their performance characteristics that can only be determined through a rigorous assessment of sensitivity, specificity, positive/negative predictive value and pre-test probabilities of active infection in a given population.

### History of COVID-19 and Other Coronaviruses That May Impact Virus Testing

**Figure 1:**
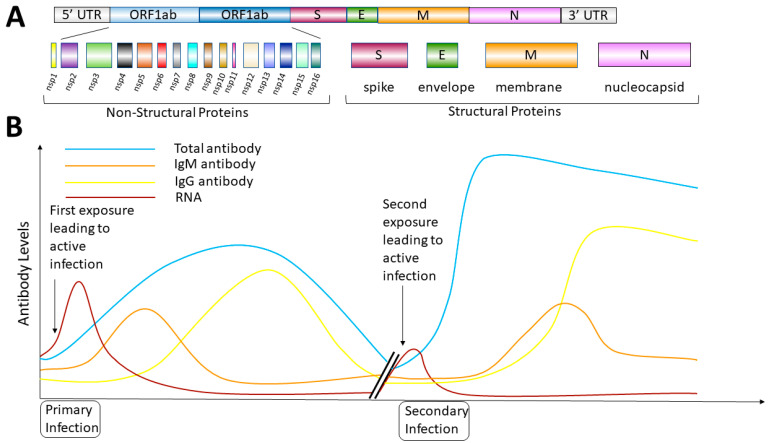
The SARS-CoV-2 Genome and Antibody Responses (**A**). SARS-CoV-2 RNA genome (30 kB) and its coding regions encoding both non-structural and structural proteins. (**B**). Theoretical antibody responses in humans following both primary and possible secondary infections with SARS-CoV-2 and associated variants.

At this time, there have been several distinct coronaviruses discovered that infect humans and cause disease. Four mainly cause mild respiratory illness (229E, OC43, NL63 and HKU1) and three can cause more severe disease (SARS-CoV-1, MERS-CoV-1 and SARS-CoV-2) in a higher percentage of patients [16]. SARS-CoV-1 was discovered in 2003 and was the first coronavirus that frequently caused severe respiratory illness while also binding to the ACE2 receptor for virus entry. However, SARS-CoV-1 was limited in its spread globally mostly to China and Hong Kong [17]. Another coronavirus was discovered in 2003 and named NL63. This coronavirus also uses the ACE2 to the receptor for entry; however, this virus usually only causes mild respiratory illness and spreads similarly to other viruses that cause the common cold [18]. The more recently discovered SARS-CoV-2 is an enveloped, positive-strand RNA virus (Figure 1A) that can cause severe respiratory illness and also uses the ACE2 receptor to facilitate virus entry [19]. Importantly, it also has spread globally in a similar fashion as other common cold coronaviruses but with a higher propensity to cause severe disease [20], and it has more recently demonstrated the ability to mutate into new viral variants that can replace previously identified dominant strains in a given population.

With regards to coronaviruses epidemiology in general, large-scale comprehensive screening efforts focused on the spread of common-cold causing coronaviruses, have provided useful insight into the current pandemic. A study published in 2010 interrogated the four common- cold coronaviruses by analysis of 11,661 diagnostic respiratory samples from all age groups, collected in the United Kingdom, between July 2006 and June 2009 [21]. It was reported that individuals are exposed and seroconvert from infection with these common cold coronaviruses in childhood. Now many adults, for the first time, are being exposed to the coronavirus that is SARS-CoV-2. Infection with common cold coronaviruses is common, including 229E and OC43, that were discovered in the 1950s and 1960s, and possibly cause reinfection due to waning immunity. Newer coronaviruses that also cause mild respiratory illness include NL63 and HKU1, and they likely also cause repeated infections [22]. Importantly, during the development of antibody tests in 2003 for SARS-CoV-1, cross-reactivity was reported between SARS-CoV-1 and samples containing either 229E or OC43 [23]. However, these non-specific tests can be improved upon through the use of multiplex assays and methods, including western blot, that may offer more specificity; importantly, this underscores the complexity of testing for coronavirus infection [21].

## 2. Epidemiologic Data

The U.S. is currently an epicenter of the global pandemic, with over 48 million cases and approximately 770,000 deaths with this information accessed on the 23 of November, 2021 (https://covid.cdc.gov/covid-data-tracker), and the arrival of viral variants are contributing to recent increases in infections and viral breakthrough in vaccinated individuals and deaths. However, many states in the U.S. are moving forward to resume normal activities in businesses and schools while ramping up large gathering events, including sporting activities and live music concerts. It is anticipated that these numbers will continue to rise at predictable intervals for the foreseeable future (https://covid19.healthdata.org/united-states-of-america?view=infections-testing&tab=trend&test=infections, accessed on 23 November 2021) [24]. The U.S. is currently testing approximately 0.5 to 3 million individuals daily, including both asymptomatic [25] and symptomatic patients, for COVID-19 (https://coronavirus.jhu.edu/testing, accessed on 23 November 2021). Data suggests that the U.S. should endeavor to test 3–4 million individuals per day to be able to diagnose, isolate and quarantine appropriately to mitigate the continued growth of the pandemic. Sporadic cases of reinfection with SARS-CoV-2 and associated viral variants further complicate these testing efforts [26].

### 2.1. SARS-CoV-2 Molecular Characteristics

The genome of the virus consists of a 30-kilobase RNA genome (Figure 1A) [27]. The 5′ region encodes its non-structural proteins, and its structural proteins are encoded toward the 3′ untranslated region [28]. Upon binding of SARS-CoV-2 to its receptor ACE2, it is internalized and uncoats following acidification in endocytic vesicles [29]. This acidification and uncoating are important for the virus to be able to release its genomic RNA into the cytoplasm. Once released, since the genomic RNA is positive stranded, the RNA genome can be directly translated into its viral proteins after host ribosomes bind the 5′ region [30].

The viral genomic RNA encodes specific structural proteins, including the envelope (E), membrane (M), nucleocapsid (N) and the spike (S) protein. This S protein is the specific viral protein through which this virus attaches to cells and enters through interactions with the ACE2 receptor. The S glycoprotein is expressed on the outer surface of the envelope that surrounds an inner nucleocapsid that is a ribonucleoprotein. This N protein is important for interacting with the viral genome and is produced in high abundance [31].

It is important to point out that SARS-CoV-2 encodes multiple proteins that can generate antibody responses. A common target is the S glycoprotein, and many vaccine strategies are targeting this specific viral protein [32]. As a consequence, it is imperative that future antibody tests are generated that target antibodies to other viral proteins, including the N protein, that is produced in a large quantity by SARS-CoV-2 [33]. This will allow a test to distinguish between patients that are vaccinated against the virus S protein and those individuals that have been naturally infected with SARS-CoV-2 but are unvaccinated or those vaccinated individuals that have experienced viral breakthrough.

Figure 1B is a diagram depicting a theoretical antibody response profile of someone that has been infected with SARS-CoV-2 [34]. When an individual is first exposed to SARS-CoV-2, the viral RNA becomes detectable and over time, the patient subsequently generates both IgM and IgG antibodies that may control the virus and lead to a decrease in circulating viral genomes to undetectable levels [35]. If a second infection is encountered with the same virus that has not undergone a significant antigenic shift, then a more robust antibody response may be produced that should more quickly control this second infection [36]. However, we do not have rigorous evidence if this happens in all patients exposed to SARS-CoV-2, but it can be used to understand antibody testing and the possibility of reinfection and viral breakthrough in those that are vaccinated [37].

Importantly, antibody testing in immunocompromised individuals can be more complex [38]. There is evidence that antibody responses are impaired in older individuals [39], in persons living with HIV and in other immunocompromised populations [40]. Both B and T-cell compartments are adversely affected in these individuals, and testing may be needed a second time several weeks after an initial test, if it is negative, to confirm that the patient is indeed antibody negative. In addition, multiple testing approaches will most probably be needed to appropriately characterize immune responses in these individuals. Determining the ability to generate lasting antibody responses and ascertaining prevalence data from the community, especially in individuals vaccinated for SARS-CoV-2, will be important in understanding the scale of the pandemic, future vaccine utility and prospects for achieving functional herd immunity either through vaccination or natural infection [41].

### 2.2. COVID-19 Symptoms

At this time, testing for active COVID-19 infection (nucleic acid or antigen) is primarily being done in individuals with symptoms, those with known exposures, in healthcare settings and as surveillance in high-risk environments, including schools and nursing homes, and also in athletes [42]. Since there is a higher risk of poor clinical outcomes in individuals that are older in age and that have serious chronic health conditions, it is important to test these patient populations when COVID-19 is suspected [43]. Signs and symptoms of COVID-19 include those associated with other respiratory viruses, including influenza; however, some symptoms affect other organ systems in COVID-19. Typical symptoms of respiratory virus infection include fever/chills, cough, shortness of breath, difficulty breathing, fatigue, muscle/body aches, headache, sore throat and congestion or runny nose. Moreover, COVID-19 specific symptoms may include new onset of loss of taste/smell, nausea/vomiting and diarrhea as well as Multisystem inflammatory syndrome in children (MIS-C) with COVID-19 [44]. Given that many of these symptoms can overlap with influenza and respiratory syncytial virus (RSV) infection, it is important to also test for these viruses, especially during flu season [42,45], and many companies, including Cepheid, are now offering convenient multiplex testing for as many as four respiratory viruses at one time [46].

### 2.3. Time Range of Infectious Period and Clearance

At this time, ten to fourteen days is the standard for an appropriate quarantine period for COVID-19 to ensure the minimal spread of the virus based on viral load measurements and symptomatic presentation [47]. Therefore, the overall testing window can be two to twelve days following exposure. Optimally, testing can be considered five to seven days following exposure, with seven days post exposure being favored. Patients usually present with symptoms two to five days following exposure [48] and can be virus positive one to three days before symptom onset; therefore, it is better to conduct testing as soon as symptoms arise, or as close to a known exposure as possible, so as not to progress too far from the day of exposure (https://www.cdc.gov/coronavirus/2019-ncov/symptoms-testing/symptoms.html, accessed on 23 November 2021). If an individual has had symptoms and has recovered, it may be more appropriate to test for antibodies to the N protein, with testing available from LabCorp in the U.S., to determine if an individual was indeed infected with SARS-CoV-2 as opposed to testing for active infection through a nucleic acid or antigen test [42].

### 2.4. Diagnostic Testing Overview

The U.S. Food and Drug Administration (FDA) heavily regulates diagnostic testing to diagnose viral infections. For device and test kit manufacturers, obtaining FDA diagnostic testing approval usually involves a long process of validation and comparison studies. Similarly, clinical laboratories that seek testing licensure must meet rigorous standards. Due to the public health need with the current pandemic, many SARS-CoV-2 diagnostic tests have been approved by the FDA for emergency use authorization (EUA) after limited validation studies have been conducted [49]. This has led to a wide range in performance characteristics when different tests are compared. Notably, the FDA EUA status of a particular test is considered temporary, only being valid during the time period associated with the national health emergency in the U.S. After the emergency, the FDA reserves the right to revoke FDA EUA approval, requiring the manufacturer to perform additional studies to obtain full FDA approval.

In addition to manufacturers, during the first several months of the COVD-19 pandemic, licensed clinical reference laboratories and hospital-based laboratories submitted applications for FDA EUA approval for “laboratory-developed” SARS-CoV-2 tests. In order to decompress the overwhelming demand for EUA test reviews, the FDA permitted high-complexity CLIA-certified laboratories to perform validations of their internally-developed SARS-CoV-2 tests as Laboratory Developed Tests (LDTs). Overall quality and performance characteristics are defined by the laboratory accrediting agency, such as the College of American Pathologists (CAP) in the U.S.

There can also be problems with testing, not only based on the characteristics of the test but also with the sample that is obtained. Considerations for sample procurement include ensuring that the sample is appropriate and adequate to contain sufficient viral material to be detected by the assay being employed [50]. Additionally, the sensitivity and specificity of the test must be considered; however, due to obtaining EUA approval, adequate information may not be available on a given SARS-CoV-2 test when compared to FDA-approved tests that are used to diagnose other viruses [51]. With time, only testing using the most rigorous approaches in the future will likely continue. Currently, the medical community is dependent on testing from facilities that have appropriate infrastructure to conduct adequate testing; however, it is difficult to ensure the rigor and reproducibility that is available in all testing laboratories, with the plethora of tests for SARS-CoV-2, that is observed with testing for other viruses [12]. In addition, the lack of availability of standardized testing reagents and kits, that have achieved the more rigorous standard FDA approval, also contributes to these challenges with testing [49,51].

### 2.5. General Virus Testing Approaches

There are several approaches used to test for SARS-CoV-2 [52]. Molecular testing involves the detection of viral nucleic acid, after amplification, and test results can determine whether or not a patient has an active infection that may be transmissible depending on the viral load. Most technologies utilize Polymerase Chain Reaction (PCR) that requires temperature variation and can take longer from sample input to result. PCR is a frequently used molecular method for viral nucleic acid detection due to its high sensitivity. Importantly, PCR-based tests are also more amenable to providing semi-quantitative results pertaining to viral load [53]. Newer, non-PCR based methods utilize approaches that can facilitate the rapid identification of nucleic acids using technologies leveraging isothermal amplification [54,55]. These tests are more amenable to use in the point-of-care setting given that they have the ability to provide a rapid, qualitative result. Molecular testing is costlier due to the need to utilize delicate nucleic acid polymerases, heat-labile enzymes that drive the amplification reactions needed for sample detection [56]. Early in the pandemic, there were no genotyping or variant-specific tests needed due to the lack of functional variation in the SARS-CoV-2 genome. Variants are encountered with other viruses, including HIV and HCV, and viral genotyping assays are frequently utilized in the testing and clinical management of these chronic viral infections. Furthermore, it does not appear that the first-generation SARS-CoV-2 antivirals (e.g., remdesivir) select for drug-resistant SARS-CoV-2 mutants, which are highly transmissible [57,58]. However, that may change with new oral antivirals being made available in the fourth quarter of 2021. Unfortunately, distinct SARS-CoV-2 variants have been identified and are driving new infections globally. As a result, variant-specific tests are now being developed and utilized. Importantly, since variant testing is not the primary aim of diagnostic testing for COVID-19 in general, standard testing approaches that provide reproducible results by targeting both conserved sequences within the genome and antigenic regions in viral proteins are most desired.

An additional approach utilized to diagnose active infection involves technologies that are capable of detecting viral antigens [59,60]. These tests can rapidly detect various viral proteins, including the SARS-CoV-2 S and N, and the results can be read and documented by smartphones to report test results to interested parties. Viral antigen testing is standard practice for influenza and RSV testing and is usually done with samples obtained from a nasopharyngeal swab, but it can also be performed on blood samples [59,61]. These antigen tests tend to also be less expensive than molecular assays given the lower cost of the associated reagents for this testing platform. However, the sensitivity of this technique is lower when compared to other methods for SARS-CoV-2 testing that utilize an amplification step. Interestingly, this lower sensitivity may be useful in screening efforts, especially in asymptomatic individuals, since “low” positive PCR test results may indicate an infection in an individual unlikely to be able to spread the virus to others.

In addition to approaches to diagnose active virus infection, there are also serologic testing approaches [33]. Typical serologic tests focus on the detection of human antibodies recognizing viral proteins, and these include IgM, IgG (Figure 1B) or total antibody levels [62]. These antibody tests provide insight as to whether or not an individual has been exposed to SARS-CoV-2 or vaccinated against the S protein [63]. These tests can be lower in cost and they can also be amenable to rapid point-of-care testing from either blood or saliva. However, the generation of these tests takes longer because they require biological reagents, including viral proteins antigens, and also capture antibodies. This differs from nucleic acid testing, where PCR primers can be generated very quickly and are very specific for a distinct viral genome. Table 1 describes the utilization of these tests in the clinical evaluation of a patient that is suspected to be infected with SARS-CoV-2.

## 3. Anatomic Testing Site

It is important to note that for testing for SARS-CoV-2, the site of sample acquisition is a large determinant of test performance. For viral nucleic acid testing, sensitivity can vary greatly. In symptomatic patients, nasopharyngeal swabs are more sensitive (63%) than oropharyngeal swabs (32%) while bronchoalveolar lavage fluid specimens are the most sensitive (93%). It appears that SARS-CoV-2 may move from the upper airway to the lower airway with disease progression that accompanies the presence of more severe symptoms. Testing samples from multiple sites may improve sensitivity and reduce false-negative results. Risks of false negatives and testing turnaround time are also important considerations. Testing patients with clear symptoms of COVID-19 infection can improve test performance with adequate sample acquisition. Saliva testing is also being used more routinely since sample processing strategies have been developed that improve nucleic acid release from virions while also utilizing proteinase K to process more viscous samples [64].

### 3.1. Molecular Testing-(Multi-Step vs. One-Step) and (Quantitative vs. Qualitative)

For molecular testing, there are a variety of platforms currently being used [65]. The simplest nucleic acid tests involve only a few steps, including sample acquisition straight to sample analysis and subsequent test results [64]. These tests can be quantitative, as is the molecular PCR-based test run on the Cepheid GeneXpert (45 min-PCR), or qualitative, as from the Abbott ID Now (15 minute-isothermal). PCR tests can take longer to perform given the need for multiple cycles at different temperatures, but the result can yield semi-quantitative results by providing a cycle threshold (CT) that can be useful clinically in making decisions to prevent nosocomial spread in healthcare settings [66].

In addition to these simple tests, other EUA-approved diagnostic assays may require additional separate processing steps that include viral RNA isolation and cDNA synthesis in addition to the standard amplification and detection steps [65]. The addition of sample processing steps increases the time needed to complete the test and can also be a source of variation in test performance. In addition, these multiple steps can be automated or done manually, adding additional variation in test performance [65]. A newer EUA-approved platform is among the most sensitive and uses droplet digital (dd)PCR that can detect five units/copies of SARS-CoV-2 in one milliliter for a partitioned sample [67]. However, to achieve a lower limit of detection, this platform requires multiple sample processing steps and is more expensive to perform when compared to standard PCR-based testing [14].

### 3.2. Antigen Tests

SARS-CoV-2 viral antigen tests have received EUA approval, and this is a significant addition to the testing capabilities available in the U.S. [60]. Antigen testing has been used routinely for many other viruses, including influenza and RSV from nasopharyngeal samples, and denotes active infection [59,61]. Since there is no amplification process, antigen detection is amenable to rapid point-of-care testing in diverse settings, including nursing homes and assisted living facilities. Although the sensitivity of these tests may not be as high as with molecular tests, they usually are lower in cost to perform. In addition, given that the sensitivity is not as high, they are amenable for use in screening programs since individuals that are antigen-positive may have corresponding higher viral loads at the time of testing and may also be more infectious and therefore require isolation and/or quarantine [68]. For negative testing results on symptomatic patients in healthcare settings, a reflex molecular test should be performed due to the lower sensitivity of antigen tests (Table 1). There are several antigen testing platforms available from established companies including Becton Dickinson, Abbott and Quidel, and they are straightforward sample-in result-out platforms.

### 3.3. Serology/Antibody Tests

Antibody tests are frequently used in the diagnosis of viral infections, especially as a lower-cost option to screen for chronic viral infections. These tests can detect antibodies that may only bind viral proteins or also specifically neutralize the S protein to prevent virus entry by binding its receptor-binding domain (RBD) [69]. The potential utilities of antibody tests are numerous [33]. They can facilitate the detection of SARS-CoV-2 in recently infected patients who present late in their disease course, with very low viral loads beneath the detection limit of molecular assays [70]. This is also important when lower respiratory tract sampling is not possible because upper airway secretions, including saliva, may not contain as much viral RNA as seen in the lower respiratory tract of infected individuals as the disease progresses (Table 1). By identifying the presence of these antibodies, the identification of potential convalescent plasma donors can also be accomplished [71]. It will also allow for the verification of functional vaccine responses, which is an area of need, once antibody correlates of protection are indeed verified, especially those involving S antibody levels. Antibody testing may also support the identification of healthcare workers that have some protection from future infection through the presence of neutralizing antibodies since it is very important to limit the nosocomial spread of SARS-CoV-2 [33,63,66]. Importantly, as we think about chronic illnesses in the setting of COVID-19 disease, these antibody tests can support the identification of patients that may have future disease exacerbations of chronic organ-specific illnesses following exposure and resolution to SARS-CoV-2 infection particularly when chronic lung disease is present before infection [72].

There are also potential drawbacks to these serologic assays if they are not well-validated before use [62]. False negatives may be found if performed early in the disease course, as IgM may only develop as early as one week after exposure. False negatives, in this case, result from patients that were tested too early before they have developed detectable antibody responses. They may also occur in patients that only developed a very mild disease that did not progress systemically and was limited only to the upper airway. False positives are also a risk, particularly with tests for IgM due to potential cross-reactivity with common cold coronaviruses, that were already mentioned [21]. Moreover, if SARS-CoV-2 spike protein continues to be the target of many vaccines, this will necessitate testing for other viral antigens, including the N protein, in the future, to distinguish between vaccinated and naturally exposed patients, as previously mentioned.

Antibody testing platforms that are currently being used include lateral flow assays that utilize chromatographic strips for testing [73]. This is commonly used in tests from companies including Orasure that have tests available for both HIV and HCV antibody testing. These CLIA-waived tests are performed individually as point-of-care tests, and they can give results in less than 20 min. These tests can be performed in the community or as home-based tests, but they tend to be higher in cost (approximately USD 20 per test) when using this methodology [74,75]. An additional platform uses the ELISA technology, which is more amenable to high-throughput screening [52,70]. Screening using the ELISA platform can be even lower in cost, at approximately USD 5 per test. Both of these tests only require serum or plasma, but ELISA can be done in an automated high-throughput format. There are automated instruments in many CLIA-certified laboratories available that can be used for antibody testing, and these solid-phase tests can be performed even in a higher throughput, low-cost workflow by licensed technicians.

As was mentioned previously, antibody tests may also be designed to detect the presence of neutralizing antibodies that can prevent virus entry through direct binding to the S protein RBD [70]. Neutralization assays can be used as possible surrogates of immune protection in clinical studies and may be performed in diagnostic laboratories. In vitro neutralizing assays utilize serum or plasma from persons following infection and can leverage multiple experimental parameters, including both inhibition of viral entry and viral replication in cell culture [50]. These virus-neutralizing tests (VNT) can incorporate the use of infectious SARS-CoV-2 virions if appropriate laboratory safety protection measures are available [62]. The plaque reduction neutralization test (PRNT) specifically uses SARS-CoV-2 or recombinant virus expressing reporter proteins, and these tests can take several days to complete [50]. Those that use pseudotyped viruses (e.g., vesicular stomatitis virus or lentiviruses), which express the S protein, can be performed in laboratories with standard blood-borne pathogen safety procedures (e.g., BSL-2 laboratories) and are categorized as pseudovirus neutralization tests (pVNT). In general, virus-based neutralization assays are currently not authorized for emergency use by the FDA. Finally, competitive neutralization tests (cVNT) have also been developed, that can be performed in BSL-2 laboratories, and one has been authorized under an EUA. These are antibody affinity tests designed to qualitatively detect potentially neutralizing antibodies, typically those that prevent interaction of a reporter-fused RBD with the ACE2 receptor, in an ELISA format [62].

Overall, the antibody response targeting SARS-CoV-2 in infected patients remains relatively uncharacterized for both breadth and potency, especially for differences between variants, and research is currently underway to clarify this issue [34,36]. Differences in the generation of neutralizing antibodies against SARS-CoV-2 between individuals have been observed, both in older and immunocompromised persons, and these differences are likely important. Both false positives and negative test results remain a concern in commercially available tests with EUA designation in the U.S. [70]. Importantly, serologic tests have now been developed from established companies and these tests will likely have higher sensitivity and specificity than previous tests that were available from newer companies with limited experience in generating FDA-approved viral tests.

### 3.4. SARS-CoV-2 Variant Analysis

Since the start of the pandemic, SARS-CoV-2 has acquired numerous mutations, resulting in the generation of a number of functional variants that have emerged from the original Wuhan-strain RNA genome [6]. Some of these new strains have been classified as variants of concern (VOC) by the Centers for Disease Control and Prevention (CDC) and the World Health Organization (WHO) based on evidence linked to conserved mutations (e.g., D614G, L452R, P681R, T478K, E484K/Q, E156-F157del) predominantly in the spike protein. It is thought that these mutations may impact important viral functional characteristics, including increased infectivity, and an enhanced ability to evade natural infection and/or vaccine-induced neutralizing antibody responses. For some of these variants, progressive increase in viral fitness may arise from newly acquired mutations, and variants can be found, de novo, in immunosuppressed individuals with long COVID-19 [3]. The focus has been previously placed on four currently circulating VOC in the U.S. These include (1) Alpha (B.1.1.7), first detected in the United Kingdom (UK) in September 2020; (2) Beta (B.351, B.1.351.2, B.1.351.3), emerged in South Africa in late 2020; (3) Delta (B.1.617.2, AY.1, AY.2, AY.3, AY.3.1), first detected India in early 2021; and (4) Gamma (P.1, P.1.1, P.1.2), initially reported in Japan and later identified in Brazil in December 2020. However, since its emergence, the Delta variant has more recently outcompeted other VOC, becoming the dominant strain in many countries including the U.S. [6] Newer variants have been discovered, including Omicron (B.1.1.529), Mu (B.1.621) and C.1.2, but it is unknown whether these variants will spread globally, as the Delta variant has. Interestingly, the Omicron (B.1.1.529) variant has been reported to have an increased number of mutations in its S gene when compared to the established VOCs that appeared earlier in 2021.

While next-generation sequencing (NGS) is recognized as the gold standard for SARS-CoV-2 variant identification and characterization of mutations in the viral genome [76], it is neither practical nor sustainable for most virus-testing laboratories. Specifically, NGS for SARS-CoV-2 can be cost- and labor-intensive. In addition, the turnaround time for results can take as much as one week to be finalized given the complex workflow for the analysis of sequence data, which may require more advanced computing approaches and large computer memory space. In contrast, PCR-based approaches have been a simpler approach to study specific VOC. A commercially available reverse transcriptase quantitative polymerase chain reaction (RT-qPCR) diagnostic test (TaqPath COVID-19 Combo Kit, Thermo Fisher) has become a useful PCR-based test for tracking the Alpha (B.1.1.7) variant. This was enabled by the discovery that a deletion (H69-V70) in the spike (S) protein resulted in S gene target amplification failure (SGTF), while target amplification of the nucleocapsid (N) and open reading frame (ORF) 1ab gene targets were unaffected [77]. Importantly, the newly described Omicron (B.1.1.529) variant also contains the H69-V70 deletion rendering the TaqPath testing kit useful for detecting this variant in settings where the Alpha (B.1.1.7) variant is not a major component of the variant pool. However, NGS verification is always advised, as multiple other variants can develop the H69-V70 deletion independently.

Given this specific example, in conjunction with NGS verification, RT-qPCR can serve as a cost-effective, rapid method to monitor variant prevalence using amplification of characteristic mutations. Moreover, since the Delta variant currently comprises most of the infected samples, sequencing can become redundant. Validation of samples as Delta by PCR can support sequencing efforts to be focused on new variants that may displace Delta in the future, including Omicron (B.1.1.529). However, in addition to confirming RT-qPCR-based surveillance results, NGS serves to monitor the emergence and dynamics of novel SARS-CoV-2 variants, as well as the presence of key mutations, like E484K, which are known to be present in the Beta (B.1.351) and Gamma (P.1) lineages but independently acquired in a number of Alpha variant genomes [6]. Furthermore, precise monitoring of variant pool dynamics can serve in the early detection of emerging novel viral strains. Considering the dynamics of viral mutation and the speed with which new VOC emerge, continuous surveillance of non-Delta variants is imperative through both PCR and sequencing-based approaches. This was underscored by the rapid increase of Delta, and consequent displacement of the previously dominant Alpha (B.1.1.7) and Gamma (P.1) variants in the U.S that was facilitated by the Alpha (B.1.1.7) targeted PCR-based test described above [77].

## 4. Conclusions

Given the rapid emergence and spread of the COVID-19 pandemic, the global diagnostic community has rapidly and efficiently developed testing strategies to detect many components of the SARS-CoV-2 virion and its associated variants. When compared to other pandemics, the global efforts to develop and improve testing capabilities for this deadly virus have been unparalleled [12]. However, in the U.S., the need for rapid assay development and subsequent approval through the attainment of an EUA has superseded the traditional long and arduous diagnostic testing approval workflow mandated by the FDA. It is anticipated that technology development to facilitate testing for COVID-19 will positively impact diagnostic capabilities for other viruses. For example, there is still no FDA-approved point-of-care nucleic acid or antigen test for Hepatitis C, which is the most common chronic bloodborne infection in the U.S. In less than one year, these tests have been developed for COVID-19 and have received EUA approval. Clearly, these efforts were supported by the availability of increased resources from multiple parties, including governments, the private sector and diagnostic companies, which include those that were not traditionally involved in virus testing before this pandemic. All of these efforts are contributing to the steady increase in the ability to test for SARS-CoV-2 faster, cheaper and with increased accuracy. It is anticipated that the U.S. will be able to further increase and sustain its testing capability to approximately 100 million per month, which will support efforts to keep the economy open while limiting the spread and subsequent poor clinical outcomes associated with COVID-19 in susceptible populations.

## Figures and Tables

**Table 1:** Testing workflow for the SARS-CoV-2 virus in patients suspected of having active infection.

SARS-CoV-2 Testing Algorithm					
#1 Signs and Symptoms		#2 Diagnostic Testing		#3 Follow-Up	
Primary	Additional	Rapid	Conventional	Positive Test Result	Negative Test Result
Recent onset of acute respiratory symptoms including: Sore throat Cough Shortness of breath	Fever Diarrhea Vomiting Recent loss of smell or taste Chills Muscle Fatigue	Isothermal nucleic acid amplification assay Antigen detection assay Viral sequencing assay	One-Step or Multiple-Step RT-PCR assay In Positive Samples, RT-PCR for viral variants with confirmation by sequencing	***Subsequent Monitoring*** Report positive findings following reporting guidelines Emphasize prevention measures to limit spread (isolation/quarantine) Consider therapeutic intervention for more severe symptoms	***Consider Possible False Negative Result*** Exposure history Other clinical findings Antigen test was performed Perform antibody test if available to document possible exposure

## References

[1] Randall R.E., Griffin D.E. (2017). Within host RNA virus persistence: Mechanisms and consequences. Curr. Opin. Virol..

[2] Weiss S.R., Leibowitz J.L. (2011). Coronavirus pathogenesis. Adv. Virus Res..

[3] Burke M.J., Del Rio C. (2021). Long COVID has exposed medicine′s blind-spot. Lancet Infect. Dis..

[4] Gao H., Yao H., Yang S., Li L. (2016). From SARS to MERS: Evidence and speculation. Front. Med..

[5] Kiyuka P.K., Agoti C.N., Munywoki P.K., Njeru R., Bett A., Otieno J.R., Otieno G.P., Kamau E., Clark T.G., van der Hoek L. (2018). Human Coronavirus NL63 Molecular Epidemiology and Evolutionary Patterns in Rural Coastal Kenya. J. Infect. Dis..

[6] Corey L., Beyrer C., Cohen M.S., Michael N.L., Bedford T., Rolland M. (2021). SARS-CoV-2 Variants in Patients with Immunosuppression. N. Engl. J. Med..

[7] Liu W., Fontanet A., Zhang P.H., Zhan L., Xin Z.T., Baril L., Tang F., Lv H., Cao W.C. (2006). Two-year prospective study of the humoral immune response of patients with severe acute respiratory syndrome. J. Infect. Dis..

[8] Wu L.P., Wang N.C., Chang Y.H., Tian X.Y., Na D.Y., Zhang L.Y., Zheng L., Lan T., Wang L.F., Liang G.D. (2007). Duration of antibody responses after severe acute respiratory syndrome. Emerg. Infect. Dis..

[9] Chen J., Subbarao K. (2007). The immunobiology of SARS. Annu. Rev. Immunol..

[10] Hope-Simpson R.E., Golubev D.B. (1987). A new concept of the epidemic process of influenza A virus. Epidemiol. Infect..

[11] Chau C.H., Strope J.D., Figg W.D. (2020). COVID-19 Clinical Diagnostics and Testing Technology. Pharmacotherapy.

[12] Katsarou K., Bardani E., Kallemi P., Kalantidis K. (2019). Viral Detection: Past, Present, and Future. Bioessays.

[13] Dirlikov E., Fechter-Leggett E., Thorne S.L., Worrell C.M., Smith-Grant J.C., Chang J., Oster A.M., Bjork A., Young S., Perez A.U. (2020). CDC Deployments to State, Tribal, Local, and Territorial Health Departments for COVID-19 Emergency Public Health Response—United States, January 21-July 25, 2020. MMWR Morb. Mortal. Wkly. Rep..

[14] Mostafa H.H., Hardick J., Morehead E., Miller J.A., Gaydos C.A., Manabe Y.C. (2020). Comparison of the analytical sensitivity of seven commonly used commercial SARS-CoV-2 automated molecular assays. J. Clin. Virol..

[15] Karthik K., Aravindh Babu R.P., Dhama K., Chitra M.A., Kalaiselvi G., Alagesan Senthilkumar T.M., Raj G.D. (2020). Biosafety Concerns During the Collection, Transportation, and Processing of COVID-19 Samples for Diagnosis. Arch. Med. Res..

[16] Li S.W., Lin C.W. (2013). Human coronaviruses: Clinical features and phylogenetic analysis. Biomedicine.

[17] Lai M.M.C. (2003). SARS virus: The beginning of the unraveling of a new coronavirus. J. Biomed. Sci..

[18] Pyrc K., Jebbink M.F., Berkhout B., van der Hoek L. (2004). Genome structure and transcriptional regulation of human coronavirus NL63. Virol. J..

[19] Mittal A., Manjunath K., Ranjan R.K., Kaushik S., Kumar S., Verma V. (2020). COVID-19 pandemic: Insights into structure, function, and hACE2 receptor recognition by SARS-CoV-2. PLoS Pathog..

[20] Tzotzos S.J., Fischer B., Fischer H., Zeitlinger M. (2020). Incidence of ARDS and outcomes in hospitalized patients with COVID-19: A global literature survey. Crit. Care.

[21] Gaunt E.R., Hardie A., Claas E.C., Simmonds P., Templeton K.E. (2010). Epidemiology and clinical presentations of the four human coronaviruses 229E, HKU1, NL63, and OC43 detected over 3 years using a novel multiplex real-time PCR method. J. Clin. Microbiol..

[22] Rucinski S.L., Binnicker M.J., Thomas A.S., Patel R. (2020). Seasonality of Coronavirus 229E, HKU1, NL63, and OC43 From 2014 to 2020. Mayo Clin. Proc..

[23] Lee H.K., Lee B.H., Seok S.H., Baek M.W., Lee H.Y., Kim D.J., Na Y.R., Noh K.J., Park S.H., Kumar D.N. (2010). Production of specific antibodies against SARS-coronavirus nucleocapsid protein without cross reactivity with human coronaviruses 229E and OC43. J. Vet. Sci..

[24] Marsland R., Mehta P. (2020). Data-driven modeling reveals a universal dynamic underlying the COVID-19 pandemic under social distancing. medRxiv.

[25] Huff H.V., Singh A. (2020). Asymptomatic transmission during the COVID-19 pandemic and implications for public health strategies. Clin. Infect. Dis..

[26] To K.K., Hung I.F., Ip J.D., Chu A.W., Chan W.M., Tam A.R., Fong C.H., Yuan S., Tsoi H.W., Ng A.C. (2020). COVID-19 re-infection by a phylogenetically distinct SARS-coronavirus-2 strain confirmed by whole genome sequencing. Clin. Infect. Dis..

[27] Wang H., Li X., Li T., Zhang S., Wang L., Wu X., Liu J. (2020). The genetic sequence, origin, and diagnosis of SARS-CoV-2. Eur. J. Clin. Microbiol. Infect. Dis..

[28] Ortiz-Prado E., Simbana-Rivera K., Gomez-Barreno L., Rubio-Neira M., Guaman L.P., Kyriakidis N.C., Muslin C., Jaramillo A.M.G., Barba-Ostria C., Cevallos-Robalino D. (2020). Clinical, molecular, and epidemiological characterization of the SARS-CoV-2 virus and the Coronavirus Disease 2019 (COVID-19), a comprehensive literature review. Diagn. Microbiol. Infect. Dis..

[29] Datta P.K., Liu F., Fischer T., Rappaport J., Qin X. (2020). SARS-CoV-2 pandemic and research gaps: Understanding SARS-CoV-2 interaction with the ACE2 receptor and implications for therapy. Theranostics.

[30] Romano M., Ruggiero A., Squeglia F., Maga G., Berisio R. (2020). A Structural View of SARS-CoV-2 RNA Replication Machinery: RNA Synthesis, Proofreading and Final Capping. Cells.

[31] Kandeel M., Ibrahim A., Fayez M., Al-Nazawi M. (2020). From SARS and MERS CoVs to SARS-CoV-2: Moving toward more biased codon usage in viral structural and nonstructural genes. J. Med. Virol..

[32] Khalaj-Hedayati A. (2020). Protective Immunity against SARS Subunit Vaccine Candidates Based on Spike Protein: Lessons for Coronavirus Vaccine Development. J. Immunol. Res..

[33] Lee C.Y., Lin R.T.P., Renia L., Ng L.F.P. (2020). Serological Approaches for COVID-19: Epidemiologic Perspective on Surveillance and Control. Front. Immunol..

[34] Hueston L., Kok J., Guibone A., McDonald D., Hone G., Goodwin J., Carter I., Basile K., Sandaradura I., Maddocks S. (2020). The Antibody Response to SARS-CoV-2 Infection. Open Forum Infect. Dis..

[35] Chen Y., Tong X., Li Y., Gu B., Yan J., Liu Y., Shen H., Huang R., Wu C. (2020). A comprehensive, longitudinal analysis of humoral responses specific to four recombinant antigens of SARS-CoV-2 in severe and non-severe COVID-19 patients. PLoS Pathog..

[36] Varnaite R., Garcia M., Glans H., Maleki K.T., Sandberg J.T., Tynell J., Christ W., Lagerqvist N., Asgeirsson H., Ljunggren H.G. (2020). Expansion of SARS-CoV-2-Specific Antibody-Secreting Cells and Generation of Neutralizing Antibodies in Hospitalized COVID-19 Patients. J. Immunol..

[37] Secchi M., Bazzigaluppi E., Brigatti C., Marzinotto I., Tresoldi C., Rovere-Querini P., Poli A., Castagna A., Scarlatti G., Zangrillo A. (2020). COVID-19 survival associates with the immunoglobulin response to the SARS-CoV-2 spike Receptor Binding Domain. J. Clin. Investig..

[38] Wei J., Zhao J., Han M., Meng F., Zhou J. (2020). SARS-CoV-2 infection in immunocompromised patients: Humoral versus cell-mediated immunity. J. Immunother. Cancer.

[39] Cunha L.L., Perazzio S.F., Azzi J., Cravedi P., Riella L.V. (2020). Remodeling of the Immune Response With Aging: Immunosenescence and Its Potential Impact on COVID-19 Immune Response. Front. Immunol..

[40] Visser L.G. (2012). The immunosuppressed traveler. Infect. Dis. Clin. N. Am..

[41] Fontanet A., Cauchemez S. (2020). COVID-19 herd immunity: Where are we?. Nat. Rev. Immunol..

[42] Hanson K.E., Caliendo A.M., Arias C.A., Englund J.A., Lee M.J., Loeb M., Patel R., El Alayli A., Kalot M.A., Falck-Ytter Y. (2020). Infectious Diseases Society of America Guidelines on the Diagnosis of COVID-19. Clin. Infect. Dis..

[43] Wiersinga W.J., Rhodes A., Cheng A.C., Peacock S.J., Prescott H.C. (2020). Pathophysiology, Transmission, Diagnosis, and Treatment of Coronavirus Disease 2019 (COVID-19): A Review. JAMA.

[44] Ferreira-Santos D., Maranhao P., Monteiro-Soares M. (2020). Covidcids. Identifying common baseline clinical features of COVID-19: A scoping review. BMJ Open.

[45] Li Y., Wang J., Wang C., Yang Q., Xu Y., Xu J., Li Y., Yu X., Zhu H., Liu J. (2020). Characteristics of respiratory virus infection during the outbreak of 2019 novel coronavirus in Beijing. Int. J. Infect. Dis..

[46] Leung E.C., Chow V.C., Lee M.K., Tang K.P., Li D.K., Lai R.W. (2021). Evaluation of the Xpert Xpress SARS-CoV-2/Flu/RSV Assay for Simultaneous Detection of SARS-CoV-2, Influenza A and B Viruses, and Respiratory Syncytial Virus in Nasopharyngeal Specimens. J. Clin. Microbiol..

[47] Nagler A.R., Goldberg E.R., Aguero-Rosenfeld M.E., Cangiarella J., Kalkut G., Monahan C.R., Cerfolio R.J. (2020). Early Results from SARS-CoV-2 PCR testing of Healthcare Workers at an Academic Medical Center in New York City. Clin. Infect. Dis..

[48] Lauer S.A., Grantz K.H., Bi Q., Jones F.K., Zheng Q., Meredith H.R., Azman A.S., Reich N.G., Lessler J. (2020). The Incubation Period of Coronavirus Disease 2019 (COVID-19) From Publicly Reported Confirmed Cases: Estimation and Application. Ann. Int. Med..

[49] Ravi N., Cortade D.L., Ng E., Wang S.X. (2020). Diagnostics for SARS-CoV-2 detection: A comprehensive review of the FDA-EUA COVID-19 testing landscape. Biosens. Bioelectron..

[50] Mawaddah A., Gendeh H.S., Lum S.G., Marina M.B. (2020). Upper respiratory tract sampling in COVID-19. Malays. J. Pathol..

[51] Mitchell S.L., St George K., Rhoads D.D., Butler-Wu S.M., Dharmarha V., McNult P., Miller M.B. (2020). Understanding, Verifying, and Implementing Emergency Use Authorization Molecular Diagnostics for the Detection of SARS-CoV-2 RNA. J. Clin. Microbiol..

[52] Behera B.C., Mishra R.R., Thatoi H. (2020). Recent biotechnological tools for diagnosis of corona virus disease: A review. Biotechnol. Prog..

[53] Smith E., Zhen W., Manji R., Schron D., Duong S., Berry G.J. (2020). Analytical and Clinical Comparison of Three Nucleic Acid Amplification Tests for SARS-CoV-2 Detection. J. Clin. Microbiol..

[54] Sidoti F., Bergallo M., Costa C., Cavallo R. (2013). Alternative molecular tests for virological diagnosis. Mol. Biotechnol..

[55] James A.S., Alawneh J.I. (2020). COVID-19 Infection Diagnosis: Potential Impact of Isothermal Amplification Technology to Reduce Community Transmission of SARS-CoV-2. Diagnostics.

[56] Lai C.C., Wang C.Y., Ko W.C., Hsueh P.R. (2020). In vitro diagnostics of coronavirus disease 2019: Technologies and application. J. Microbiol. Immunol. Infect..

[57] Yip C.C.Y., Sridhar S., Lau J.H.N., Cheng A.K.W., Leung K.H., Chen J.H.K., Chan K.H., Cheng V.C.C., Yuen K.Y. (2020). Comparative performance of two commercial sample-to-result systems for hepatitis C virus quantitation and genotyping. Expert Rev. Mol. Diagn..

[58] Zhao J., Chang L., Wang L. (2019). Nucleic acid testing and molecular characterization of HIV infections. Eur. J. Clin. Microbiol. Infect. Dis..

[59] Kanwar N., Hassan F., Nguyen A., Selvarangan R. (2015). Head-to-head comparison of the diagnostic accuracies of BD Veritor System RSV and Quidel(R) Sofia(R) RSV FIA systems for respiratory syncytial virus (RSV) diagnosis. J. Clin. Virol..

[60] Dinnes J., Deeks J.J., Adriano A., Berhane S., Davenport C., Dittrich S., Emperador D., Takwoingi Y., Cunningham J., Beese S. (2020). Rapid, point-of-care antigen and molecular-based tests for diagnosis of SARS-CoV-2 infection. Cochrane Database Syst Rev..

[61] Leonardi G.P., Wilson A.M., Mitrache I., Zuretti A.R. (2015). Comparison of the Sofia and Veritor Direct Antigen Detection Assay Systems for Identification of Influenza Viruses from Patient Nasopharyngeal Specimens. J. Clin. Microbiol..

[62] Theel E.S., Slev P., Wheeler S., Couturier M.R., Wong S.J., Kadkhoda K. (2020). The Role of Antibody Testing for SARS-CoV-2: Is There One?. J. Clin. Microbiol..

[63] Shields A., Faustini S.E., Perez-Toledo M., Jossi S., Aldera E., Allen J.D., Al-Taei S., Backhouse C., Bosworth A., Dunbar L.A. (2020). SARS-CoV-2 seroprevalence and asymptomatic viral carriage in healthcare workers: A cross-sectional study. Thorax.

[64] Smyrlaki I., Ekman M., Lentini A., Rufino de Sousa N., Papanicolaou N., Vondracek M., Aarum J., Safari H., Muradrasoli S., Rothfuchs A.G. (2020). Massive and rapid COVID-19 testing is feasible by extraction-free SARS-CoV-2 RT-PCR. Nat. Commun..

[65] Loeffelholz M.J., Tang Y.W. (2020). Laboratory diagnosis of emerging human coronavirus infections—The state of the art. Emerg. Microbes Infect..

[66] Rhee C., Baker M., Vaidya V., Tucker R., Resnick A., Morris C.A., Klompas M., for the CDC Prevention Epicenters Program (2020). Incidence of Nosocomial COVID-19 in Patients Hospitalized at a Large US Academic Medical Center. JAMA Netw. Open.

[67] Suo T., Liu X., Feng J., Guo M., Hu W., Guo D., Ullah H., Yang Y., Zhang Q., Wang X. (2020). ddPCR: A more accurate tool for SARS-CoV-2 detection in low viral load specimens. Emerg. Microbes Infect..

[68] Guglielmi G. (2020). Fast coronavirus tests: What they can and can′t do. Nature.

[69] Morrison B.J., Labo N., Miley W.J., Whitby D. (2015). Serodiagnosis for tumor viruses. Semin. Oncol..

[70] Ejazi S.A., Ghosh S., Ali N. (2020). Antibody detection assays for COVID-19 diagnosis: An early overview. Immunol. Cell Biol..

[71] Wooding D.J., Bach H. (2020). Treatment of COVID-19 with convalescent plasma: Lessons from past coronavirus outbreaks. Clin. Microbiol. Infect..

[72] Zhao Y.M., Shang Y.M., Song W.B., Li Q.Q., Xie H., Xu Q.F., Jia J.L., Li L.M., Mao H.L., Zhou X.M. (2020). Follow-up study of the pulmonary function and related physiological characteristics of COVID-19 survivors three months after recovery. EClinicalMedicine.

[73] Pickering S., Betancor G., Galao R.P., Merrick B., Signell A.W., Wilson H.D., Kia Ik M.T., Seow J., Graham C., Acors S. (2020). Comparative assessment of multiple COVID-19 serological technologies supports continued evaluation of point-of-care lateral flow assays in hospital and community healthcare settings. PLoS Pathog..

[74] Pant Pai N., Balram B., Shivkumar S., Martinez-Cajas J.L., Claessens C., Lambert G., Peeling R.W., Joseph L. (2012). Head-to-head comparison of accuracy of a rapid point-of-care HIV test with oral versus whole-blood specimens: A systematic review and meta-analysis. Lancet Infect. Dis..

[75] Smith B.D., Teshale E., Jewett A., Weinbaum C.M., Neaigus A., Hagan H., Jenness S.M., Melville S.K., Burt R., Thiede H. (2011). Performance of premarket rapid hepatitis C virus antibody assays in 4 national human immunodeficiency virus behavioral surveillance system sites. Clin. Infect. Dis..

[76] Nasir J.A., Kozak R.A., Aftanas P., Raphenya A.R., Smith K.M., Maguire F., Maan H., Alruwaili M., Banerjee A., Mbareche H. (2020). A Comparison of Whole Genome Sequencing of SARS-CoV-2 Using Amplicon-Based Sequencing, Random Hexamers, and Bait Capture. Viruses.

[77] Kidd M., Richter A., Best A., Cumley N., Mirza J., Percival B., Mayhew M., Megram O., Ashford F., White T. (2021). S-Variant SARS-CoV-2 Lineage B1.1.7 Is Associated With Significantly Higher Viral Load in Samples Tested by TaqPath Polymerase Chain Reaction. J. Infect. Dis..

